# Visual Cues Predictive of Behaviorally Neutral Outcomes Evoke Persistent but Not Interval Timing Activity in V1, Whereas Aversive Conditioning Suppresses This Activity

**DOI:** 10.3389/fnsys.2021.611744

**Published:** 2021-03-05

**Authors:** Kevin J. Monk, Simon Allard, Marshall G. Hussain Shuler

**Affiliations:** ^1^Solomon H. Snyder Department of Neuroscience, The Johns Hopkins University School of Medicine, Baltimore, MD, United States; ^2^Kavli Neuroscience Discovery Institute, Baltimore, MD, United States

**Keywords:** primary visual cortex, interval timing, trace conditioning, persistent activity, valence

## Abstract

Cue-evoked persistent activity is neural activity that persists beyond stimulation of a sensory cue and has been described in many regions of the brain, including primary sensory areas. Nonetheless, the functional role that persistent activity plays in primary sensory areas is enigmatic. However, one form of persistent activity in a primary sensory area is the representation of time between a visual stimulus and a water reward. This “reward timing activity”—observed within the primary visual cortex—has been implicated in informing the timing of visually cued, reward-seeking actions. Although rewarding outcomes are sufficient to engender interval timing activity within V1, it is unclear to what extent cue-evoked persistent activity exists outside of reward conditioning, and whether temporal relationships to other outcomes (such as behaviorally neutral or aversive outcomes) are able to engender timing activity. Here we describe the existence of cue-evoked persistent activity in mouse V1 following three conditioning strategies: pseudo-conditioning (where unpaired, monocular visual stimuli are repeatedly presented to an animal), neutral conditioning (where monocular visual stimuli are paired with a binocular visual stimulus, at a delay), and aversive conditioning (where monocular visual stimuli are paired with a tail shock, at a delay). We find that these conditioning strategies exhibit persistent activity that takes one of three forms, a sustained increase of activity; a sustained decrease of activity; or a delayed, transient peak of activity, as previously observed following conditioning with delayed reward. However, these conditioning strategies do not result in visually cued interval timing activity, as observed following appetitive conditioning. Moreover, we find that neutral conditioning increases the magnitude of cue-evoked responses whereas aversive conditioning strongly diminished both the response magnitude and the prevalence of cue-evoked persistent activity. These results demonstrate that cue-evoked persistent activity within V1 can exist outside of conditioning visual stimuli with delayed outcomes and that this persistent activity can be uniquely modulated across different conditioning strategies using unconditioned stimuli of varying behavioral relevance. Together, these data extend our understanding of cue-evoked persistent activity within a primary sensory cortical network and its ability to be modulated by salient outcomes.

## Introduction

Cue-evoked persistent activity can be defined as neural activity which persists beyond the presentation of a sensory cue. One such example of cue-evoked persistent activity is reward timing activity in rodent V1, wherein neurons produce a representation of time between a transient visual stimulus and a delayed water reward (Shuler and Bear, 2006; Chubykin et al., 2013; Monk et al., 2020). This reward timing activity takes one of three forms, each of which would qualify as cue-evoked persistent activity: a sustained increase of activity until the expected reward time, a sustained decrease of activity until the expected reward time, or a peak of activity around the expected reward time. V1 reward timing activity can also be classified as an interval timing signal having fulfilled key tenets of such a signal (as reviewed in Hussain Shuler, 2016). Although V1 reward timing activity has been extensively studied, the conditions conducive to V1 producing cue-evoked persistent activity, let alone interval timing activity, in varying conditioning strategies remain unknown. Specifically, two open questions are (1) Does cue-evoked persistent activity exist in V1 in the absence of conditioning with a delayed outcome? and (2) Is any outcome—not exclusively a rewarding outcome—sufficient to manipulate cue-evoked persistent activity within V1 (e.g., by conditioning interval timing signals)?

Cue-evoked persistent activity in the primary visual cortex to unpaired visual stimuli (i.e., visual stimuli that are not temporally related to outcomes) has been observed as sustained membrane depolarizations in mouse V1 (Funayama et al., 2015, 2016), emitted spikes in mouse V1 (Funayama et al., 2015), and complex V1 LFP responses in the mouse (Funayama et al., 2016), rat (Kimura, 1962; Zold and Hussain Shuler, 2015) and rabbit (Bishop and O’Leary, 1936). Furthermore, such responses to unpaired visual stimuli are modulated based on the stimulus’ familiarity to the animal. Specifically, previous studies have shown that V1 neurons have a larger magnitude response to familiar stimuli relative to unfamiliar stimuli (Frenkel et al., 2006; Cooke et al., 2015). Furthermore, as it pertains to the temporal domain, when visual stimuli are familiar, evoked LFP activity within V1 expresses prolonged bouts of theta oscillations to more intense visual stimulation compared to less intense stimulation (Zold and Hussain Shuler, 2015). Such findings suggest that cue-evoked persistent activity can exist in V1 outside of temporal relationships to outcomes, but it is not clear how such activity is expressed at the single-neuron level.

V1 reward timing activity is an example of interval timing activity and the ability for V1 to learn this timing activity is dependent on the release of acetylcholine (ACh) from the basal forebrain (Chubykin et al., 2013; Liu et al., 2015). When cholinergic axons are lesioned locally in V1, neurons in V1 are unable to learn reward timing activity (Chubykin et al., 2013). Furthermore, local application of the ACh agonist, carbachol, is sufficient to engender—that is, give rise to—reward-timing-like activity in V1 slice recordings (Chubykin et al., 2013). Additionally, paring visual stimuli with delayed, optogenetic activation of basal forebrain fibers or cholinergic fibers in V1 is sufficient to engender interval timing activity within mouse V1 (Liu et al., 2015). Targeted recordings of basal forebrain cholinergic neurons (BFCNs) show strong responses to salient events such as water rewards and electric shocks and weaker responses to sensory stimuli such as auditory cues (Hangya et al., 2015; Guo et al., 2019). As a range of outcomes can activate BFCNs and their activation is sufficient to engender timing activity within V1, it is possible that pairing visual cues to non-rewarding outcomes (e.g., aversive shocks or neutral visual stimuli) can influence the timing and/or prevalence of cue-evoked persistent activity within V1 (where prevalence here is defined as the proportion of neurons expressing cue-evoked persistent activity across the population).

Here, we investigate these two open questions: whether cue-evoked persistent activity requires temporal relationships to outcomes and whether non-rewarding outcomes can affect cue-evoked persistent activity in V1. We find that V1 neurons express persistent activity to unpaired, familiar visual stimuli and that the forms this activity takes are the forms observed during V1 reward timing. We also demonstrate that neither neutral nor aversive outcomes are able to engender timing activity within V1 neurons as previously demonstrated for rewarding outcomes. However, we find that neutral conditioning significantly increases neural response magnitude whereas aversive conditioning strongly diminishes the prevalence of cue-evoked persistent activity demonstrating that conditioning strategies which vary in their behavioral relevance differentially influence sensory-evoked responses within cortical circuits.

## Materials and Methods

All procedures reported here were in accordance with the US NIH Guide for the Care and Use of Laboratory Animals and were approved by the Animal Care and Use Committee at the Johns Hopkins University School of Medicine.

### Animal Information and Surgical Procedures

A total of 13 male, C57BL/6 (Strain Code: 027, Charles River Laboratories) mice (aged between 2 and 6 months; weight range: 20–30 g) were used in this study. Ten mice underwent pseudo-conditioning prior to undergoing neutral conditioning (*n* = 4) or aversive conditioning (*n* = 6). Additionally, three mice underwent neutral conditioning without experiencing pseudo-conditioning. Surgical procedures are as previously described (Monk et al., 2020) and are recapitulated here.

Surgical procedures were performed under aseptic conditions and were in accordance with the Animal Care and Use Committee at the Johns Hopkins School of Medicine. Animals underwent two surgeries spaced at least 2 weeks apart from one another. Prior to either surgery, mice were anesthetized using a cocktail of ketamine (Ketaset, 80 mg/kg) and xylazine (Anased, 10 mg/kg) and eyes were covered with ophthalmic ointment (Puralube). The first surgery was performed to affix a head-restraint bar to the animal’s skull for training purposes and to mark sites for future craniotomies. In the first surgery, the hair covering the skull was removed (Nair), the skin cleaned with alternating 70% ethanol and iodine, then the skin was cut away. Following this, the periosteum was removed and the skull cleaned with alternating 70% ethanol and hydrogen peroxide, then the skull was dried with canned air. A total of four sites were marked for future craniotomies: two for ground screws (arbitrarily marked over the anterior parietal bone) and two for primary visual cortex (measured as 3 mm lateral to lambda, bilaterally). Sites for future craniotomies were covered in a silicone elastomer (Smooth-On Body Double) and a head-post was affixed to the anterior portion of the mouse’s skull with super glue (Loctite 454). The remaining bone was covered in super glue. A second surgery was performed to implant recording electrodes. Briefly, small craniotomies were made using a dental drill for ground screws and screws were implanted into sites. Next, craniotomies were created over V1, the dura was cleaned with sterile paper points, and electrodes were brought to the surface of the brain, then implanted 500 μm below the cortical surface in accordance with stereotaxic measurements of V1 (Franklin and Paxinos, 2008). Wires were covered in sterile ophthalmic ointment (Puralube) and encased in dental cement (Orthojet). Ground screws and ground wires were connected and a headcap was built of dental cement.

### Pseudo-Conditioning Task

**FIGURE 1:**
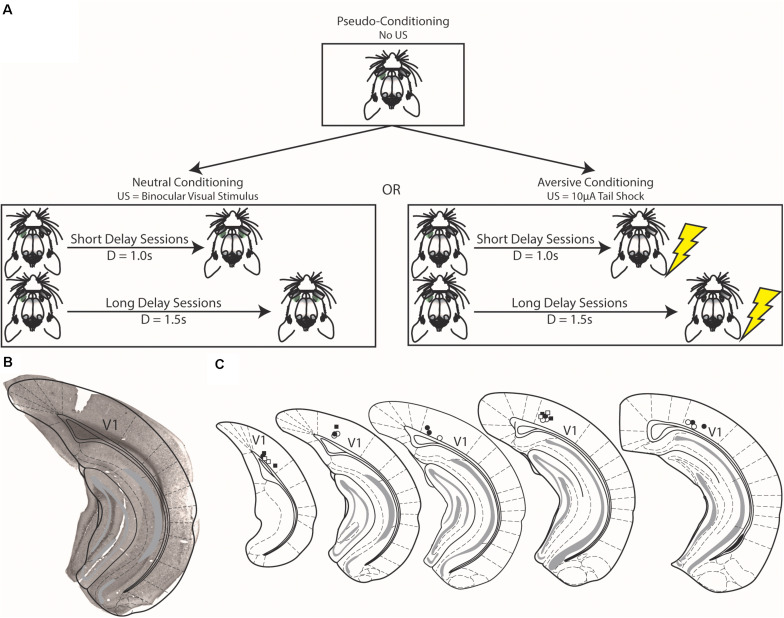
Schematics of conditioning strategies and histological verification of electrode placement. **(A)** In pseudo-conditioning, unpaired visual stimuli were presented to animals for a number of days **(top)**. After which, visual stimuli were either paired with either a binocular visual stimulus [Neutral Conditioning, **(left)**] or a 10 μA tail shock [Aversive Conditioning, **(right)**]. The final forms of the task are shown in this figure, but preceding the final instantiations, animals were shaped as described in the text. **(B)** Example brain section from experimental mouse showing electrode implant is confined to the primary visual cortex (V1). **(C)** Schematic demonstrating electrode placement across all animals. Squares represent electrode placement for animals that underwent aversive conditioning and circles represent animals that underwent neutral conditioning; filled shapes denote implants from the left hemisphere and open shapes denote implants from the right hemisphere. Schematic adapted from Franklin and Paxinos (2008).

Following electrode implantation, animals recovered from surgery for 5–7 days after which they were habituated to head fixation over the course of 2 days before experiencing unpaired visual stimuli. Monocular visual stimuli were presented to mice as previously described (Monk et al., 2020). Briefly, these cues were presented to animals via head-mounted, custom-made goggles placed over the animal’s eyes. These goggles consist of a miniature LED glued to the back of a translucent, plastic hemidome. Visual cues were 100 ms, full-field retinal flashes of light. These cues are the same used in alternate conditioning strategies (see below), and during pseudo-conditioning, they were presented with equal probability (in a pseudo-random fashion) to either the left or right eye. Animals underwent 3–5 days of pseudo-conditioning prior to moving on to either neutral or aversive conditioning (Figure 1A, and described below). Additionally, a subset of trials (20%) were “sham” trials in which no cue was delivered.

### Neutral and Aversive Conditioning Tasks

Animals either underwent neutral conditioning or aversive conditioning (Figure 1A). Seven animals underwent neutral conditioning (four of which underwent pseudo-conditioning prior to neutral conditioning) and six animals underwent aversive conditioning (all of which underwent pseudo-conditioning prior to aversive conditioning). These two conditioning strategies are largely the same and are similar to pseudo-conditioning (see above). In neutral conditioning, monocular visual cues were presented pseudo-randomly with equal probability to the left or right eye. This stimulus was paired with a delayed binocular visual stimulus (a full-field retinal flash of light delivered simultaneously to both eyes via the same head-mounted goggles as the preceding monocular visual cue). However, during aversive conditioning, monocular visual cues were paired with a delayed 10 μA electric shock delivered to the tail via custom-made tail cuffs (the strength of this stimulus was parameterized prior to conditioning, see below). Data presented here were recorded during conditioning sessions in which the delay between the monocular visual stimulus and the outcome was either 1 s (Short Delay Sessions) or 1.5 s (Long Delay Sessions) after visual stimulus offset. Note that though we use two monocular cues, the two cues predicted the same outcome at the same delay. These recording sessions occurred following a conditioning procedure that mimics conditioning of reward-interval timing that occurs during appetitive conditioning (Monk et al., 2020). Animals underwent consecutive sessions of Short and Long Delay sessions. Though we have discussed the conditioning strategies together, animals only experienced either neutral or aversive conditioning. As in pseudo-conditioning, a subset of trials (20%) were “sham” in which no cue was delivered; these trials allowed for a proxy measure of spontaneous activity within the task.

### Parameterization of Tail Shocks

When conditioning reward timing activity in V1, the rewarding outcome does not evoke activity within V1 though it does produce a behavioral response (Shuler and Bear, 2006; Chubykin et al., 2013; Monk et al., 2020). When investigating the effect aversive conditioning has on V1 responses, we sought to use an outcome that, like reward timing, did not activate V1 neurons but produced a behavioral response. As such, we parameterized the strength of tail shocks prior to conditioning in the following manner. In a separate recording room, we presented mice in the aversive conditioning blocks of 50 tail shocks of increasing magnitude starting with a block of “blank” shocks (0 μA) to determine a baseline of activity. Following blank shocks, animals experienced blocks of 50 tail shocks of the following magnitudes: 5, 10, 20, and 40 μA (in that order). Evoked activity was normalized across recorded neurons to baseline activity [via area under the ROC curve (AUC) normalization, see below] and was plotted across shock magnitude. We determined that 10 μA was the strongest stimulus to evoke a behavioral response that did not have an obvious, direct effect on evoking neural activity (data shown below). As such, we used 10 μA as the outcome in aversive conditioning. These data are described in more detail in the Results and are presented in Figure 4.

### Electrophysiology

Neural activity was recorded as previously reported (Monk et al., 2020). Neural activity was recorded bilaterally from primary visual cortex using custom-built recording electrodes. Per recording electrode, 16 channels of neural data were recorded at a sampling rate of 32,556 Hz through commercial hardware (Neuralynx). Neurons were offline identified through manual 3D cluster-cutting methods through commercial software (Offline Sorter, Plexon) and were treated as independent units across recording sessions. Electrodes composed of a connector with 16 recording channels and two ground wires (Omnetics). Bundles were cut at a ∼45° bias to allow for sampling across a depth of approximately 250 μm.

### Data Analysis

Analysis performed here is consistent with a report of reward timing activity in mouse V1 (Monk et al., 2020) and is described below. Analysis was performed using custom code in MATLAB (Mathworks). To define statistically significant differences across distributions we first tested whether one or more of the distributions were likely to come from a normal distribution family (i.e., using the Lilliefors test). Having found that at least one of the tested distributions did not likely come from a normal family (α = 0.05), we used non-parametric tests throughout this report (e.g., Friedman test, Wilcoxon rank-sum test, etc.). Results of the Lilliefors tests are presented in Supplementary Table 1.

#### Cue-Evoked Persistent Activity Classification

V1 reward timing activity is an example of cue-evoked persistent activity and takes one of three forms: a sustained increase of activity until the expected time of reward (SI), a sustained decrease of activity until the time of reward (SD), or a peak of activity around the time of reward (PK). Here, we manually classified neural responses across various conditioning strategies in a blinded fashion to determine the existence of cue-evoked persistent activity outside of reward conditioning contexts. Specifically, an individual neuron was randomly selected from all recorded sessions across all animals. Then, an AUC-normalized neural response (see below) recorded during left-cue, right-cue, or sham trials was randomly selected and presented. This neural response was then classified as “Not Classified” (NC), “Sustained Increase” (SI), “Sustained Decrease” (SD), or “Peak” (PK). SI and SD responses are classified based on the presence of a sustained increase or decrease of activity after the conditioned stimulus (CS) stimulation window, respectively; conversely, PK responses are classified based on the presence of a prominent peak of neural activity after any initial visual-evoked response. Activity from the remaining trial types were presented (in a random fashion), followed by the remaining neurons (again selected randomly). These classifications were performed without knowledge of animal identity, recording session, delay time, or conditioning strategy. This blind classification process was recently used and cross-validated to classify reward timing within mouse V1 (Monk et al., 2020).

#### Neural Report of Time Calculation

Neural reports of time (NRTs) were calculated for each neuron with persistent activity. For SI and SD response forms, the NRT is calculated as the moment when neurons return to a baseline level of activity. For PK response forms, the NRT is the time of maximum firing rate from baseline (after any initial visual-evoked response). This calculation involved first normalizing neural activity to baseline with AUC normalization and then applying an algorithmic calculation of the NRT. This calculation strategy has previously been employed to define NRTs for V1 reward timing activity (Monk et al., 2020) and the details of which are summarized below.

To calculate an NRT, neural activity was normalized to the baseline firing rate by calculating the AUC using a sliding, 100 ms window (Cohen et al., 2015; Sadacca et al., 2018). An AUC value of 0.5 means that the ideal observer would be at chance level to tell apart two distributions and values above or below 0.5 reflect greater dissimilarity among two distributions. For our purposes, we found the AUC value between the distribution of spike counts from a 100ms window of baseline pre-stimulus activity, and a given 100 ms of spiking activity across all trials of the same type. In this way, we do not rely on the averaging of spike counts in the same way that a peri-stimulus time histogram (PSTH) does and thus the resultant value is more robust against a small subset of trials with many spikes or other forms of inter-trial spiking variability. Furthermore, this method normalizes the firing rate to a value bounded by 0 and 1 for every set of trials. As the AUC-normalized firing rate is the magnitude of difference and not the sign of the difference between an AUC value and 0.5 (which determines how dissimilar two distributions are), we found the absolute value of the difference between the AUC vector and a value of 0.5. In doing so, neurons with sustained activation or suppression (SI or SD neurons, respectively) could be treated with the same algorithm to calculate an NRT. We operationally defined a difference threshold of 0.15 (true AUC value of 0.35 or 0.65), and, using this threshold, we then defined the NRT as the first moment in time when the AUC difference vector fell below the threshold for at least 100 ms. For classified PK neurons, the NRT was defined as the time of the maximum of this AUC difference vector. To avoid conflating timing responses with general visual responses, we set a minimum value for valid NRTs as 0.5 s after stimulus offset.

#### Evoked Energy Calculation

To determine whether the magnitude of evoked responses was affected by conditioning strategies, we calculated evoked energy scores of neural responses within the first 1 s following CS stimulation. Specifically, for a given neural PSTH we calculated the area between neural activity and baseline activity (defined as the median firing rate from 1 s before CS onset). Thus, we were able to quantify the magnitude of a response across the first second following CS stimulation. We have used a 1 s window as during this time window, the experimental epoch is identical across conditioning strategies. That is, the commonly shared time epoch is as follows: 0–0.1 s, a visual stimulus is delivered and from 0.1 to 1 s, no stimuli are delivered.

### Histology

To confirm recording electrode locations, standard histology procedures were undertaken. Briefly, animals were deeply anesthetized using sodium pentobarbital (200 mg/kg, Vedco). After which, animals were transcardially perfused with ice cold phosphate-buffered saline (PBS) followed by ice cold 4% paraformaldehyde (PFA). Brains were immersion fixed overnight in 4% PFA and were transferred to 30% sucrose until sectioning. Brains were sectioned on a cryostat into 60 μm slices. Electrode location was verified using Nissl staining, as follows. Sections containing V1 were selected and mounted on gelatin subbed slides and air dried. These slides were then immersed in a solution containing 0.1% Cresyl violet and 1% glacial acetic acid dissolved in water for 5 min, followed by a 2-min wash in distilled water, then by 2 min in 50% ethanol, then 2 min in 70% ethanol. Stained and washed sections were air dried, immersed in xylenes then coverslipped with Permount Mounting Medium (Electron Microscopy Sciences). Histological results are shown in Figures 1B,C.

## Results

Cue-evoked persistent activity is evoked neural activity that persists beyond stimulation with a sensory cue. One well-studied example of this activity is V1 reward timing activity that is expressed in one of three canonical forms: a sustained increase of activity until the expected time of reward (SI), a sustained decrease of activity until the time of reward (SD), and a peak of activity around the time of reward (PK). We first investigated whether similar cue-evoked persistent activity can exist when visual cues are presented in the absence of a delayed outcome by measuring activity patterns during “pseudo-conditioning” wherein monocular visual cues were presented without a delayed outcome. We then determined the effect of pairing visual cues of varying behavioral relevance has on cue-evoked activity within V1. This was done through “neutral conditioning” and “aversive conditioning” wherein monocular visual stimuli were paired with a delayed binocular visual stimulus or a 10μA tail shock, respectively.

### Cue-Evoked Persistent Activity Following Pseudo-Conditioning

**FIGURE 2:**
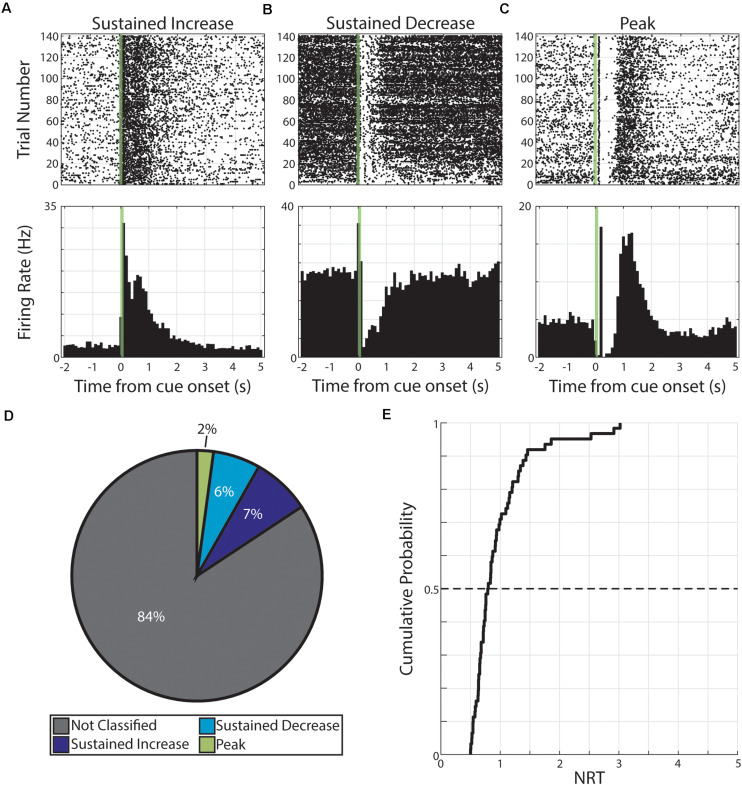
Persistent activity observed during pseudo-conditioning. **(A–C)** Three example neurons are shown expressing cue-evoked persistent activity in one of three forms: a sustained increase of activity **(A)**, a sustained decrease of activity **(B)**, or a delayed peak of activity **(C)**. Green shaded bar represents time of visual stimulation. **(D)** Pie chart showing proportion of responses classified with persistent activity and proportions of forms. **(E)** Cumulative probability plot of calculated neural reports of time (NRTs) of responses with persistent activity recorded during pseudo-conditioning.

Pseudo-conditioning, here, refers to repeated presentations of unpaired, monocular visual stimuli across several days. During this conditioning strategy, we recorded from 541 neurons across 10 animals. From this recording population, we classified a total of 1,082 neural responses (each neuron having a response to both monocular cues; 541 neurons × 2 cues = 1,082 neural responses). Of these responses, we find 170 were classified as having persistent activity. Specifically, these 170 responses were expressed by 121 neurons and the distributions of response forms are as follow: 81 responses expressed by 63 neurons were classified as SI, 66 responses expressed by 50 neurons were classified as SD, and 23 responses expressed by 21 neurons were classified as PK (Figure 2).

We then used these persistent activity patterns to determine the time course of these responses. Specifically, we defined the NRT as the time that SI and SD return to baseline activity or the time of maximum activation (following visual-evoked responses) for PK responses (see section “Materials and Methods”). We find that we can derive a distribution of NRTs for this pseudo-conditioned population of neurons (median +standard deviation = 0.80 +052 s, Figure 2). In future sections, we describe different effects that neutral and aversive conditioning have on neural populations. To confirm that any potential across-strategy differences cannot be explained by underlying differences across experimental subjects, we compared features of neural activity across what future conditioning strategy animals will undergo. Across a range of neural response features, we found no significant differences (*p* > 0.05, Wilcoxon rank-sum test or χ^2^ goodness-of-fit test; Supplementary Table 2) across neurons regardless of whether animals would undergo neutral conditioning (“Future Neutral”) or would undergo aversive conditioning (“Future Aversive”). Specifically, we tested: latency to first CS-evoked spike, firing rate outside of pseudo-conditioning trials, the number of spikes within the CS stimulation window, and the likelihood that neural response would be classified as having cue-evoked persistent activity. Full results for these comparisons can be found in Supplementary Table 2. These results suggest that there are no significant differences across experimental subjects and that any differences we observe across conditioning strategies are likely the result of the different conditioning strategies.

These pseudo-conditioning results indicate that presenting familiar visual cues in the absence of a temporal relationship to outcomes is sufficient to evoke persistent activity in V1 and that this activity takes the same forms as observed in V1 reward timing activity. This observation is consistent with prior work reporting that repeated visual cues gave rise to prolonged theta oscillations in rat V1, the duration of which initially reflects the intensity of the visual cue, only to subsequently converge to the reward delay time (Zold and Hussain Shuler, 2015). Additionally, these data indicate that in the absence of a paired outcome, cortical networks can produce persistent activity. Thus, when investigating potential instances of interval timing activity, it is necessary to change the time between events as done in previous studies (Shuler and Bear, 2006; Chubykin et al., 2013; Zold and Hussain Shuler, 2015; Monk et al., 2020) and as we have done for neutral and aversive conditioning (see below).

### Cue-Evoked Persistent Activity Following Neutral Conditioning

Cue-evoked persistent activity can occur without temporal relationships to outcomes (Figure 2). Additionally, pairing a visual stimulus with a delayed water reward affects cue-evoked activity by engendering timing activity within V1 representing the conditioned interval (Shuler and Bear, 2006; Chubykin et al., 2013; Zold and Hussain Shuler, 2015; Monk et al., 2020). We thus sought to address whether pairing visual stimuli with any delayed outcome would affect cue-evoked persistent activity or whether there is a relationship between an outcome’s potential to influence cortical activity and its behavioral relevance. We began our investigation into this question with a behaviorally neutral conditioning strategy in which monocular visual stimuli were paired with a delayed binocular visual stimulus (Figure 1A). Briefly, binocular visual stimuli followed monocular visual stimuli at either a 1 s (Short Delay Sessions) or 1.5 s (Long Delay Sessions).

**FIGURE 3:**
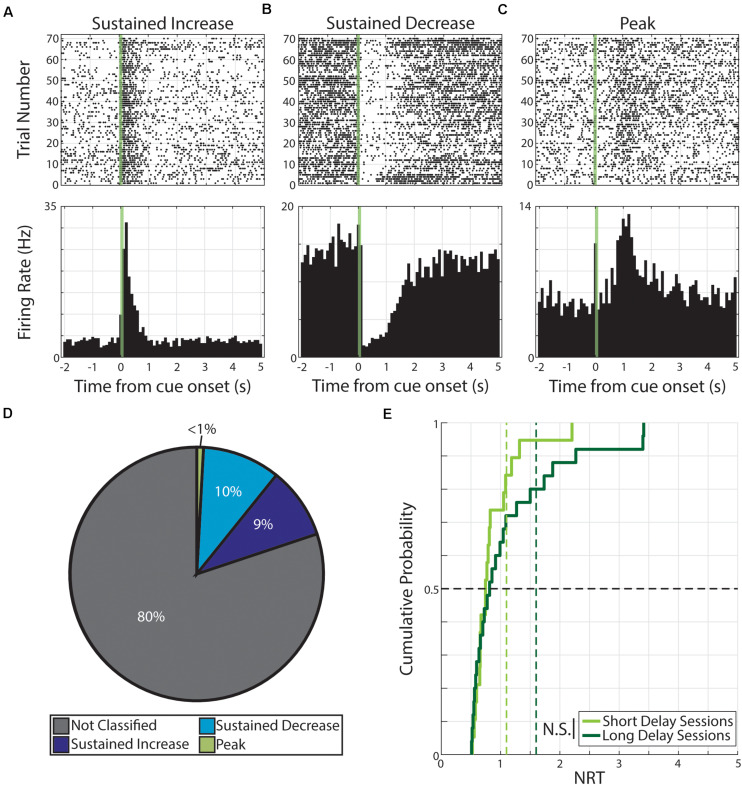
Persistent activity observed during neutral conditioning. **(A–C)** Three example neurons are shown expressing cue-evoked persistent activity in one of three forms: a sustained increase of activity **(A)**, a sustained decrease of activity **(B)**, or a delayed peak of activity **(C)**. Green shaded bar represents time of visual stimulation. **(D)** Pie chart showing proportion of responses classified with persistent activity and proportions of forms. **(E)** Cumulative probability plot of calculated neural reports of time (NRTs) of responses with persistent activity recorded during short delay sessions (light green) or long delay sessions (dark green). These distributions are not significantly different from each other. *N.S.: Not significant.*

During neutral conditioning, we recorded from 176 neurons across seven animals. As in the case with pseudo-conditioning, we find evidence of cue-evoked persistent activity. Specifically, of the 352 neural responses (176 neurons × 2 cues), we find 70 responses were classified as having persistent activity. Specifically, these 70 responses were expressed by 51 neurons and the distribution of response forms are as follow: 32 responses expressed by 24 neurons were classified as SI, 35 responses expressed by 26 neurons were classified as SD, and 3 responses expressed by 3 neurons were classified as PK (Figure 3).

**FIGURE 4:**
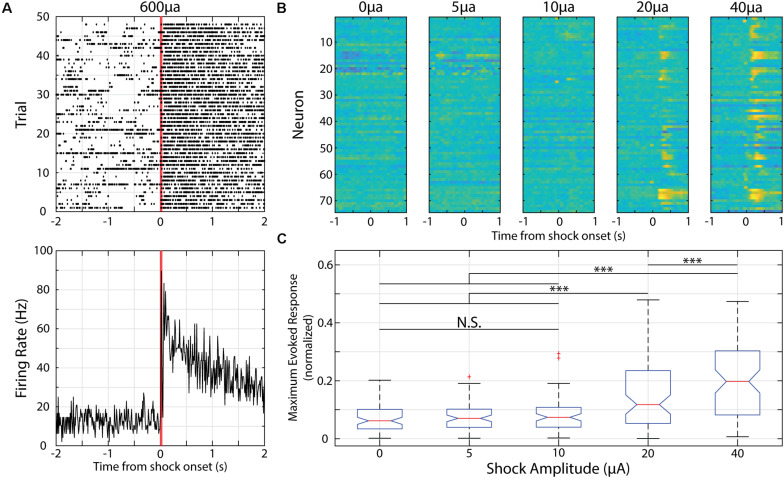
Neurons in V1 respond to electric shocks delivered to the animal’s tail of various magnitudes. **(A)** Example neural activity presented as raster plot **(top)** or PSTH **(bottom)** in response to a 30 ms, 600 μA electric shock delivered to the animal’s tail. Red lines indicate onset and offset of electric shock. **(B)** Heatmaps of recorded neurons during shock parameterization. Each row represents normalized activity recorded from a neuron during unpaired shock delivery across a range of shock amplitudes. **(C)** Box plot showing distribution of maximum evoked response from neurons within first 500 ms of shock delivery (maximum value is 0.5). ^∗∗∗^*p* < 0.001, Tukey’s HSD *post hoc* test following Friedman’s Test. N.S., Not significant.

We then calculated the NRT distributions for both Short Delay (*n* = 19 NRTs) and Long Delay Sessions (*n* = 25 NRTs) in neutral conditioning and find that the central tendencies do not accord with the conditioned intervals (Short Delay NRTs: 0.75 +0.39 s; Long Delay NRTs: 0.82 +0.82 s). Additionally, we find that the two distributions are not significantly different from each other (*Z* = −0.56, *p* = 0.58, Wilcoxon rank-sum test, Figure 3).

Animals that underwent neutral conditioning had different training histories (i.e., four underwent pseudo-conditioning, “Pseudo→Neutral”, and three underwent only neutral conditioning, “Neutral Only”). To control whether this difference in training history influenced the effects of neutral conditioning on cortical activity, we compared neural response features across individual animals. In doing so we found that both groups had similar prevalence of cue-evoked persistent activity and neither group exhibited interval timing activity (Supplementary Table 3). However, the magnitude of the visual-evoked response was significantly affected by training history (see below). These data indicate that although persistent activity is also observed in neutral conditioning (similar to responses observed in pseudo-conditioning), these responses do not qualify as “interval timing” responses as they do not adequately reflect the conditioned interval between the two events.

### Cue-Evoked Persistent Activity Following Aversive Conditioning

Having demonstrated that visual stimuli presented in the absence of a delayed outcome or when paired with a delayed, neutral outcome result in cue-evoked persistent activity, we sought to address whether a behaviorally-relevant, non-rewarding outcome would affect evoked responses. Specifically, we addressed whether paring a visual stimulus with a delayed, aversive outcome influences V1 cue-evoked persistent activity.

Electric shocks are readily used for fear conditioning and shocks delivered to a mouse’s tail is sufficient to train head-restrained mice to associate a visual stimulus with an upcoming tail shock during an active avoidance task (Makino and Komiyama, 2015). When using a shock magnitude as previously reported (i.e., 600 μA), we find strong and prolonged responses in V1 neurons (Figure 4A). As such, we parameterized the strength of the electric shock and compared the evoked responses. We find that there is a significant effect of shock magnitude on evoked responses [χ^2^(4, 369) = 67.38, *p* = 8.09 × 10^–14^, Friedman’s Test] and that a magnitude of 10 μA is the strongest to evoke a neural response that is not significantly different than spontaneous activity (as determined by responses to a “blank” 0 μA electric shock, *p* = 0.70, Tukey’s HSD test, Figure 4).

**FIGURE 5:**
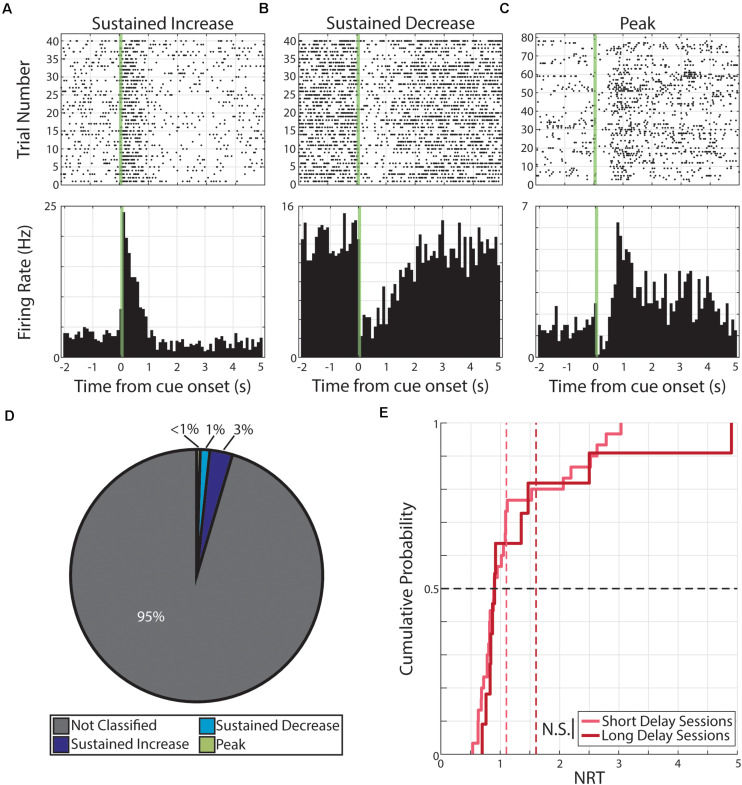
Persistent activity observed during aversive conditioning. **(A–C)** Three example neurons are shown expressing cue-evoked persistent activity in one of three forms: a sustained increase of activity **(A)**, a sustained decrease of activity **(B)**, or a delayed peak of activity **(C)**. Green shaded bar represents time of visual stimulation. **(D)** Pie chart showing proportion of responses classified with persistent activity and proportions of forms. **(E)** Cumulative probability plot of calculated neural reports of time (NRTs) of responses with persistent activity recorded during short delay sessions (light red) or long delay sessions (dark red). These distributions are not significantly different from each other. N.S., Not significant.

Upon parameterization, we then paired monocular visual stimuli with a 10 μA electric shock at a 1 s (Short Delay Sessions) or 1.5 s (Long Delay Sessions) following the visual stimulus offset. During aversive conditioning, we recorded from 861 neurons from six animals. Of the 1,722 neural responses (861 neurons × 2 cues), we find that 79 responses were classified as having persistent activity. Specifically, these 79 responses were expressed by 56 neurons and the distribution of responses forms are as follow: 50 responses expressed by 33 neurons were classified as SI, 21 responses expressed by 18 neurons were classified as SD, and 8 responses expressed by 8 neurons were classified as PK (Figure 5).

We then determined the distribution of calculated NRTs for responses recorded during Short Delay Sessions (*n* = 30 NRTs) and NRTs for responses recorded during Long Delay Sessions (*n* = 11 NRTs). We find that these distributions do not well accord with the conditioned intervals (Short Delay NRTs: 0.89 +0.72 s; Long Delay NRTs: 0.90 +1.26 s, Figure 5). Furthermore, these two distributions are not significantly different from each other (*Z* = −0.57, *p* = 0.57, Wilcoxon rank-sum test).

### Differential Effects of Conditioning Strategy on V1 Cue-Evoked Responses

**FIGURE 6:**
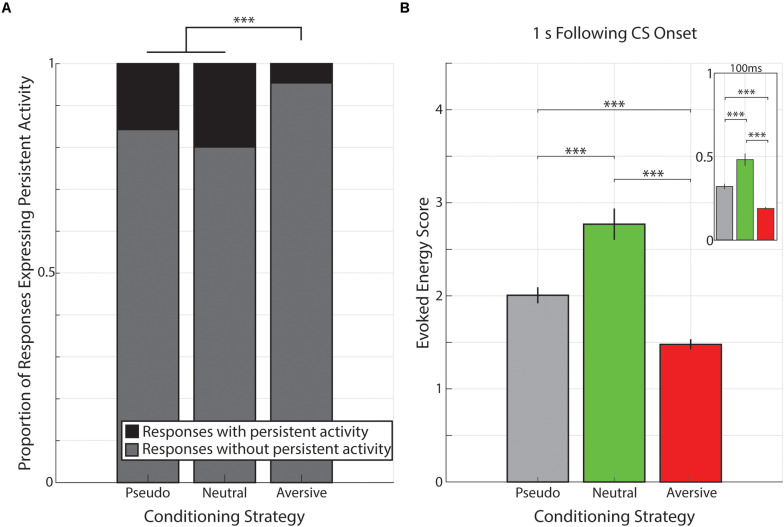
Proportion of responses with persistent activity is smaller following aversive conditioning. **(A)** Bar graph showing proportions of responses classified as expressing persistent activity (black) or not (gray) during pseudo-conditioning **(left)**, neutral conditioning **(middle)**, or aversive conditioning **(right)**. ^∗∗∗^*p* < 1 × 10^− 6^, χ^2^ goodness-of-fit test. **(B)** Bar graph demonstrating that the evoked energy of cue-evoked responses is significantly greater in animals underwent neutral conditioning and evoked energy is significantly smaller in animals that underwent aversive conditioning. Main graph shows the evoked energy calculated in the 1 s following visual stimulation and inset shows evoked energy calculated in the 100 ms of visual stimulation. Vertical bars represent mean +SEM. ^∗∗∗^*p* < 0.001, Tukey’s HSD *post hoc* test following Kruskal–Wallis Test.

The data presented thus far indicate that cue-evoked persistent activity is present across a range of conditioning strategies and that this activity is expressed in the same forms across various conditioning strategies (Supplementary Figure 1). Moreover, unlike rewarding outcomes, the non-rewarding outcomes used here are insufficient to engender cued interval timing activity in V1. However, engendering interval timing activity is not the only effect an outcome can have on cue-evoked persistent activity. Indeed, there is a significant reduction in the prevalence of cue-evoked persistent activity following aversive conditioning compared to both pseudo-conditioning [(χ^2^(1, 2804) = 101.62, *p* < 1 × 10^–6^; χ^2^ goodness-of-fit test] and neutral conditioning [χ^2^(1, 2074) = 102.58, *p* < 1 × 10^–6^; χ^2^ goodness-of-fit test]. However, there is no significant difference in the prevalence of cue-evoked persistent activity across pseudo- and neutral conditioning [χ^2^(1, 1,434) = 3.32, *p* = 0.068; χ^2^ goodness-of-fit test; Figure 6A].

Beyond the prevalence of cue-evoked persistent activity, we sought to determine whether conditioning strategies also influenced the magnitude of visual-evoked responses. To do so, we calculated the evoked energy of neural responses across all neural responses (i.e., NC, SI, SD, and PK) in the 1 s following CS onset (see Materials and Methods section) and found different values across the different conditioning strategies (presented as median +standard deviation: Pseudo-Conditioning: 1.07 +2.82, Neutral Conditioning: 1.75 +3.16, Aversive Conditioning: 0.88 +2.34; Figure 6B). We found there was a significant effect of conditioning strategy on this evoked energy score [χ^2^(2, 3153) = 106.63, *p* = 7.02 × 10^–24^, Kruskal–Wallis test). *Post hoc* analyses revealed that evoked energy scores from neural responses recorded during aversive conditioning were significantly smaller than those recorded during pseudo- or neutral conditioning (*p* = 1.47 × 10^–7^, *p* = 9.56 × 10^–10^, respectively, Tukey’s HSD) consistent with the paucity of cue-evoked persistent activity within these responses. Additionally, we found that evoked energy scores of responses recorded during neutral conditioning were significantly higher compared to those scores of responses recorded during pseudo-conditioning (*p* = 8.42 × 10^–9^, Tukey’s HSD). This pattern of evoked energy scores (i.e., Neutral > Pseudo-Conditioning > Aversive) is maintained even when analysis is limited to the 100 ms of CS stimulation (Neutral vs. Pseudo: *p* = 1.34 × 10^–5^; Neutral vs. Aversive: *p* = 9.56 × 10^–10^; Pseudo vs. Aversive: p = 9.56 × 10^–10^; Tukey HSD, Figure 6). Furthermore, when neutral conditioning animals were divided based on training history (“Pseudo→Neutral” and “Neutral Only”, see above), we find that neural responses from Pseudo→Neutral animals (1.95 +3.02) had significantly higher evoked energy scores than neural responses from Neutral Only animals (1.19 +3.39; *Z* = 3.51, *p* = 4.44 × 10^–4^, Wilcoxon rank-sum test). These results are consistent with previous mouse V1 studies which demonstrate that stimulus familiarity is sufficient to potentiate visual-evoked responses within LFP recordings (Frenkel et al., 2006; Cooke et al., 2015).

These data suggest that pairing a visual stimulus with a behaviorally-relevant outcome affects cue-evoked persistent activity by either engendering interval timing activity (as in the case of previously reported reward timing activity) and/or altering the prevalence of cue-evoked persistent activity within V1 (as seen in aversive conditioning). Furthermore, stimulus familiarity (here, repeated presentations of the same visual stimuli) can also affect neural responses independently of cue-evoked persistent activity prevalence. Together, these results demonstrate the myriad effects by which an animal’s training history can influence the visual cortex’s response to visual stimuli.

## Discussion

The work here explores the nature of cue-evoked persistent activity evoked by visual stimuli within the mouse primary visual cortex. Specifically, we investigated the requirement (or lack thereof) of a conditioned outcome to elicit such activity, whether predictable temporal relationships to delayed outcomes are sufficient to engender cued-interval timing activity, and how this activity is modulated by outcomes of varying behavioral relevance. We have shown that persistent activity can exist within mouse V1 to transient, familiar visual stimuli both in the absence of an unconditioned stimulus (pseudo-conditioning) and in the context of neutral and aversive conditioning (Figures 2, 3, 5). These data are in line with previous reports that show that extended activity within primary visual cortex occurs following presentation of familiar visual stimuli (Bigler et al., 1976; Bigler, 1977; Zold and Hussain Shuler, 2015). Additionally, while we find that neither neutral (a binocular visual stimulus) nor aversive (a 10 μA tail shock) outcomes are sufficient to engender interval timing activity in mouse V1 (Figures 3, 5), both conditioning strategies differentially influence how V1 responds to visual cues predictive of subsequent behaviorally-neutral and -aversive events (Figure 6). Neutral conditioning results in the potentiation of cue-evoked responses without influencing the prevalence of cue-evoked persistent activity (Figure 6). Conversely, aversive conditioning significantly diminishes both response magnitude and the prevalence of cue-evoked persistent activity within V1 (Figure 6). Together these results demonstrate that cue-evoked persistent activity can exist independently of temporal relationships to outcomes, and that sensory responses are subject to differential modulation based on the behavioral relevance of conditioning strategies.

### Cue-Evoked Persistent Activity in the Absence of Delayed Outcomes

We show here that when visual stimuli are presented in the absence of an unconditioned stimulus, V1 neurons express cue-evoked persistent activity (Figure 2). Such persistent activity outside of behavioral conditioning may serve to enable future learning of stimulus-outcome relationships. For instance, it has been previously demonstrated in V1 that early in training, the duration (and prevalence) of visually evoked theta oscillations correspond to the cue’s luminance, and that only with further training converge to the expected cue-reward delay (Zold and Hussain Shuler, 2015). Demonstrating the behavioral relevance of this neural activity in V1, the timing of reward-seeking actions from these visual cues covaries with oscillation duration on a trial-by-trial basis and actions are more accurate and precise when oscillations are present (Levy et al., 2017). Therefore, the presence of cue-evoked theta oscillations early in training may serve to enable future timed actions. Similarly, the presence of cue-evoked persistent activity as observed in single neurons here may promote future learning of stimulus-outcome relationships.

Additionally, the cue-evoked persistent activity we observe following pseudo-conditioning takes the same form as cue-evoked persistent activity following reward conditioning (that is, the SI, SD, and PK forms) raising the question of how these response forms arise. Previous computational models of reward timing activity posit that these forms are the result of a core network architecture that is composed of excitatory and inhibitory cells with a specific connectivity pattern (Huertas et al., 2015). We recently corroborated this computational model by showing that identified interneuron subtypes produce reward timing activity in a manner consistent with the theoretical network architecture *in vivo* (Monk et al., 2020). The presence of these forms following pseudo-conditioning suggests that this network architecture may exist within V1 independently of reinforcement and that the process of learning curates the responses of the circuit elements to aid in the production of interval timing activity as opposed to creating these responses *de novo*.

Together, these results demonstrate that cue-evoked persistent activity occurs in V1 in the absence of unconditioned stimuli. Future studies may utilize longitudinal recordings and cell-specific identifications to define the manner by which this activity enables future learning and/or the network architectures that promote these patterns of neural activity.

### Cue-Evoked Persistent Activity in the Presence of Non-Rewarding Stimuli

We show here that, unlike rewarding outcomes, pairing a visual stimulus with a neutral or aversive outcome is insufficient to engender interval timing activity within V1 (Figures 3, 5). However, we find that neutral conditioning increases the magnitude of cue-evoked responses and that aversive conditioning strongly suppresses both the magnitude of cue-evoked responses and the prevalence of cue-evoked persistent activity. Thus, conditioning visual stimuli with different outcomes uniquely affects sensory responses within cortical networks.

When the same visual stimulus is presented across several days, the magnitude of a visually-evoked neural response increases and visually-evoked behavioral movements are smaller and less frequent (Frenkel et al., 2006; Cooke et al., 2015). These familiarity-induced changes to visual responses have been argued to enable future perceptual learning (Gavornik and Bear, 2014a), and such changes have been shown to be useful in learning sequences of visual stimuli (Gavornik and Bear, 2014b). Consistent with these results, we find that the magnitude of the evoked response is greater in neurons recorded during neutral conditioning sessions. Moreover, we found that within neutral conditioned animals, those individuals with greater experience with the conditioned visual stimuli (i.e., Pseudo→Neutral animals) have stronger responses. These results demonstrate how familiarity with sensory cues can influence sensory responses in single neurons and possibly enable future learning of stimulus-outcome relationships (see above); similar studies investigating familiarity-related plasticity should carefully control for the experimental subject’s learning history.

Unlike neutral conditioning where the main effect is to strengthen the response magnitude, the main effect of aversive conditioning is to significantly reduce neural response magnitude and the prevalence of cue-evoked persistent activity within V1. Previous studies have demonstrated that in primary sensory areas, fear conditioning results in a “sparsification” of sensory responses (Gdalyahu et al., 2012; Yin et al., 2020). The network dampening caused by aversive conditioning we observe here is consistent with these previous reports.

Here, we demonstrate that conditioning with a neutral or aversive outcome differentially influences cue-evoked persistent activity. Furthermore, previous work has demonstrated that similar conditioning with a rewarding outcome engenders cued-interval timing activity within V1 (Shuler and Bear, 2006; Chubykin et al., 2013; Monk et al., 2020). How might these differing conditioning strategies uniquely affect sensory responses within cortex? Reward timing activity is dependent on the activation of BFCNs (Chubykin et al., 2013; Liu et al., 2015). Moreover, BFCNs have been shown to be activated by neutral sensory stimuli (e.g., an auditory cue), rewarding outcomes (e.g., a water reward), and aversive outcomes (e.g., electric shocks), albeit to differing degrees (Hangya et al., 2015; Guo et al., 2019). Furthermore, it has been posited that the amount of acetylcholine released within V1 would preferentially activate one receptor subtype over another (Alitto and Dan, 2013). Perhaps, the differential effects that neutral, aversive, and reward conditioning have on ongoing V1 responses are due, in part, to differential BFCN activation and, thereby, differing amounts of acetylcholine released within V1. Future studies may take advantage of recent technological advances to directly record cholinergic axons within V1 (Eggermann et al., 2014; Kuchibhotla et al., 2017) or directly from BFCNs (Hangya et al., 2015; Guo et al., 2019) to determine how conditioning with different outcomes uniquely affects cortical activity.

## Concluding Remarks

Interval timing has been shown in the rodent primary visual cortex (Shuler and Bear, 2006; Chubykin et al., 2013; Liu et al., 2015; Monk et al., 2020) and in other rodent cortical areas (Narayanan and Laubach, 2009; Parker et al., 2014; Xu et al., 2014) and is a special example of cue-evoked persistent activity. Here, we show that the expression of cue-evoked persistent activity within V1 can exist when visual stimuli are presented without delayed outcomes and that a predictable temporal relationship with a delayed outcome, alone, is not sufficient to engender cued interval timing activity. Moreover, we find that distinct outcomes differentially influence cue-evoked persistent activity. Specifically, neutral stimuli (here a second visual stimulus) can potentiate cue-evoked response magnitudes whereas aversive stimuli (here an electric shock) strongly suppress network activity. Together, these results further our understanding of cue-evoked persistent activity within cortical networks and provide insight into the manner by which behaviorally-relevant outcomes can influence ongoing network activity.

## Data Availability Statement

The raw data supporting the conclusions of this article will be made available by the authors, without undue reservation.

## Ethics Statement

The animal study was reviewed and approved by Johns Hopkins University Animal Care and Use Committee.

## Author Contributions

KM and MH designed the experiments and wrote the manuscript. KM collected and analyzed the neural data. SA and KM performed the histological verification of electrode implant strategies. All authors contributed to the article and approved the submitted version.

## Conflict of Interest

The authors declare that the research was conducted in the absence of any commercial or financial relationships that could be construed as a potential conflict of interest.
