# Supplementary figures and images for: Visual Cues Predictive of Behaviorally Neutral Outcomes Evoke Persistent but Not Interval Timing Activity in V1, Whereas Aversive Conditioning Suppresses This Activity
Source: Front Syst Neurosci. 2021 Mar 5;15:611744. doi: 10.3389/fnsys.2021.611744 (PMC7973048; doi:10.3389/fnsys.2021.611744)

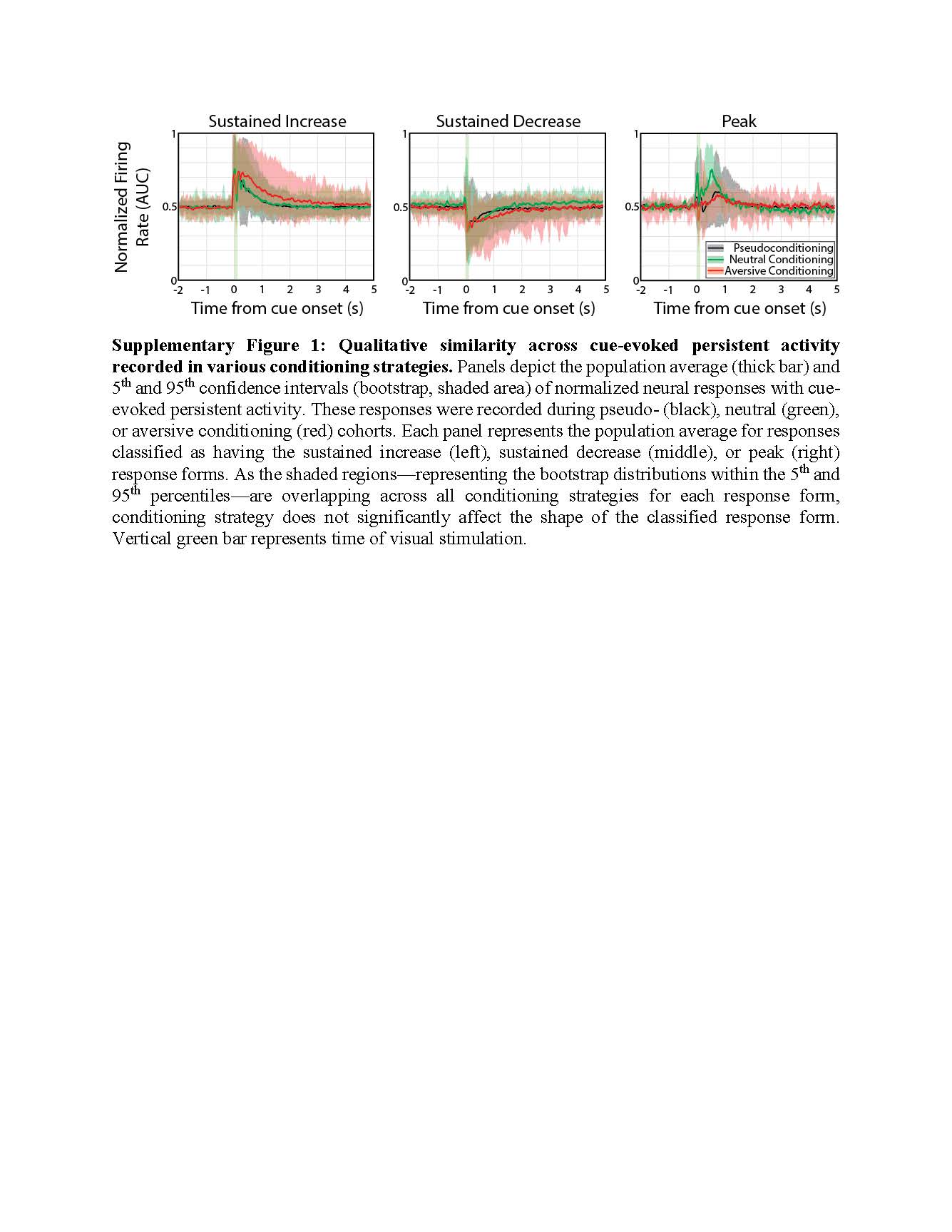

Supplement: Supplementary file 1 [file Image_1.jpg]
