# Supplementary material for: Visual Cues Predictive of Behaviorally Neutral Outcomes Evoke Persistent but Not Interval Timing Activity in V1, Whereas Aversive Conditioning Suppresses This Activity
Source: Front Syst Neurosci. 2021 Mar 5;15:611744. doi: 10.3389/fnsys.2021.611744 (PMC7973048; doi:10.3389/fnsys.2021.611744)
Supplement: Supplementary file 2 [file Table_1.pdf]

**Description of Distribution**	**Inclusive Section(s)/Figure(s)/Table(s)**	**Probability that Distribution comes from a Normal Family (Lilliefors Test)**
Future neutral; latency to first CS-evoked spike	Section 3.1, Supplemental Table 2	p = 0.04
Future aversive; latency to first CS-evoked spike	*Section 3.1, Supplemental Table 2*	*p = 0.48*
Future neutral; Firing rate outside of pseudo-conditioning trials	Section 3.1, Supplemental Table 2	p < 0.001
Future aversive; Firing rate outside of pseudo-conditioning trials	Section 3.1, Supplemental Table 2	p < 0.001
Future neutral; number of spikes within CS stimulation window	Section 3.1, Supplemental Table 2	p < 0.001
Future aversive; number of spikes within CS stimulation window	Section 3.1, Supplemental Table 2	p < 0.001
Neutral conditioning; short delay NRTs from responses classified as having cue-evoked persistent activity	Section 3.2, Figure 3	p < 0.001
Neutral conditioning; long delay NRTs from responses classified as having cue-evoked persistent activity	Section 3.2, Figure 3	p < 0.001
Pseudo→Neutral cohort; short delay NRTs from responses classified as having cue-evoked persistent activity	Section 3.2, Supplemental Table 3	p < 0.001
Pseudo→Neutral cohort; long delay NRTs from responses classified as having cue-evoked persistent activity	Section 3.2, Supplemental Table 3	p = 0.007
Neutral Only cohort; short delay NRTs from responses classified as having cue-evoked persistent activity	*Section 3.2, Supplemental Table 3*	*p = 0.50*

Neutral Only cohort; long delay NRTs from responses classified as having cue-evoked persistent activity	Section 3.2, Supplemental Table 3	$p = 0.04$
Pseudo→Neutral; All NRTs from responses classified as having cue-evoked persistent activity	Section 3.2, Supplemental Table 3	$p < 0.001$
Neutral Only; All NRTs from responses classified as having cue-evoked persistent activity	*Section 3.2, Supplemental Table 3*	*$p = 0.08$*
Evoked neural response to tail shocks	Section 3.3, Figure 4	$p < 0.001$
Aversive Conditioning; short delay NRTs from responses classified as having cue-evoked persistent activity	Section 3.3, Figure 5	$p < 0.001$
Aversive Conditioning; long delay NRTs from responses classified as having cue-evoked persistent activity	Section 3.3, Figure 5	$p = 0.003$
Evoked energy scores across conditioning strategies	Section 3.4, Figure 6	$p < 0.001$
Pseudo→Neutral cohort; evoked energy scores	Section 3.4	$p < 0.001$
Neutral Only cohort; evoked energy scores	Section 3.4	$p < 0.001$

Supplemental Table 1: Results of Lilliefors test to determine whether tested distribution is likely from Normal distribution. Column 1: description of distributions; Column 2: where distributions are presented in text, figures, and supplemental tables; Column 3: probability that a distribution comes from a normal family. A p-value less than 0.05 indicates that it is not valid to assume this distribution comes from a normal family. Here, we avoided assuming normality of tested distributions by using non-parametric tests (e.g., Wilcoxon rank-sum tests) in instances where one or more of the tested distributions (denoted here by alternating colors) was not likely from a normal family.
