# Supplementary material for: Visual Cues Predictive of Behaviorally Neutral Outcomes Evoke Persistent but Not Interval Timing Activity in V1, Whereas Aversive Conditioning Suppresses This Activity
Source: Front Syst Neurosci. 2021 Mar 5;15:611744. doi: 10.3389/fnsys.2021.611744 (PMC7973048; doi:10.3389/fnsys.2021.611744)
Supplement: Supplementary file 3 [file Table_2.pdf]

Neural Response Feature	Statistical Results	Cohort	Median + Standard Deviation
Latency to First CS-Evoked Spike	Z = -1.39, p = 0.16; Wilcoxon rank-sum test	Future Neutral	44.80 + 14.53ms
		Future Aversive	41.78 + 10.44ms
Firing Rate Outside of Pseudo-Conditioning Trials	Z = -1.79, p = 0.07; Wilcoxon rank-sum test	Future Neutral	4.18 + 6.77sp/s
		Future Aversive	5.66 + 10.18 sp/s
Number of Spikes Within CS Stimulation Window	Z = -1.68, p = 0.09; Wilcoxon rank-sum test	Future Neutral	4.41 + 7.08 spikes
		Future Aversive	6.34 + 10.58ms
Proportion of Responses Classified as Having Persistent Activity	$\chi^2 = 5.05 \times 10^{-5}$, p = 0.99; χ^2 goodness-of-fit test	Future Neutral	Proportion Classifed = 0.84
		Future Aversive	Proportion Classifed = 0.84

Supplemental Table 2: Features of neural activity within pseudo-conditioning data split by future conditioning strategies. Shown here are comparisons across different neural response features based on whether an animal underwent Neutral (Future Neutral) or Aversive (Future Aversive) conditioning after pseudo-conditioning. No significant differences were found across the range of features indicating that any across-animal differences are likely the result of differences in conditioning strategies.
