# Supplementary material for: Visual Cues Predictive of Behaviorally Neutral Outcomes Evoke Persistent but Not Interval Timing Activity in V1, Whereas Aversive Conditioning Suppresses This Activity
Source: Front Syst Neurosci. 2021 Mar 5;15:611744. doi: 10.3389/fnsys.2021.611744 (PMC7973048; doi:10.3389/fnsys.2021.611744)
Supplement: Supplementary file 4 [file Table_3.pdf]

Feature Tested	Statistical Results	Group	Median + Standard Deviation
Interval Timing Activity in Pseudo→Neutral Cohort	Z = -1.58, p = 0.11; Wilcoxon rank-sum test	Short Delay	0.71 + 0.45 s
		Long Delay	0.86 + 1.03 s
Interval Timing Activity in Neutral Only Cohort	Z = -0.85, p = 0.39; Wilcoxon rank-sum test	Short Delay	0.92 + 0.39 s
		Long Delay	0.56 + 0.32 s
All NRTs	Z = 1.24, p = 0.22; Wilcoxon rank-sum test	Pseudo→Neutral	0.78 + 0.84 s
		Neutral Only	0.66 + 0.34 s
Proportion of Responses Classified as Having Persistent Activity	χ^2 = 0.09, p = 0.77; χ^2 goodness-of-fit test	Pseudo→Neutral	Proportion Classified = 0.80
		Neutral Only	Proportion Classified = 0.81

Supplemental Table 3: Neural response features from neurons recorded during neutral conditioning split based on training history. Neurons from animals which underwent pseudo-conditioning prior to neutral conditioning ("Pseudo→Neutral") were compared to animals are compared against neurons from animals which only experienced neutral conditioning ("Neutral Only"). Over these features, no significant differences were found suggesting that training history had limited effect on most features of neural activity. However, see Section 3.4 for description of differences in evoked energy scores across neutral conditioning cohorts.
